# Telomeres and telomerase in head and neck squamous cell carcinoma: from pathogenesis to clinical implications

**DOI:** 10.1007/s10555-016-9633-1

**Published:** 2016-08-08

**Authors:** Paolo Boscolo-Rizzo, Maria Cristina Da Mosto, Enrica Rampazzo, Silvia Giunco, Annarosa Del Mistro, Anna Menegaldo, Lorena Baboci, Monica Mantovani, Giancarlo Tirelli, Anita De Rossi

**Affiliations:** 1 Section of Otolaryngology and Regional Centre for Head and Neck Cancer, Department of Neurosciences, University of Padova, Treviso, Italy; 2 Section of Oncology and Immunology, Department of Surgical Sciences, Oncology and Gastroenterology, University of Padova, via Gattamelata 64, 35128 Padova, Italy; 3 Immunology and Molecular Oncology Unit, Istituto Oncologico Veneto—IRCCS, Padova, Italy; 4 Department of Otorhinolaryngology and Head and Neck Surgery, University of Trieste, Trieste, Italy

**Keywords:** Field cancerization, Head and neck cancer, Human papillomavirus, Molecular biology, Recurrence, Survival, Telomerase reverse transcriptase, Telomere, *TERT*

## Abstract

Strongly associated with tobacco use, heavy alcohol consumption, and with high-risk human papillomavirus (HPV) infection, head and neck squamous cell carcinoma (HNSCC) is a frequently lethal, heterogeneous disease whose pathogenesis is a multistep and multifactorial process involving genetic and epigenetic events. The majority of HNSCC patients present with locoregional advanced stage disease and are treated with combined modality strategies that can markedly impair quality of life and elicit unpredictable results. A large fraction of those who undergo locoregional treatment and achieve a complete response later develop locoregional recurrences or second field tumors. Biomarkers that are thus able to stratify risk and enable clinicians to tailor treatment plans and to personalize post-therapeutic surveillance strategies are highly desirable. To date, only HPV status is considered a reliable independent predictor of treatment response and survival in patients with HNSCC arising from the oropharyngeal site. Recent studies suggest that telomere attrition, which may be an early event in human carcinogenesis, and telomerase activation, which is detected in up to 90 % of malignancies, could be potential markers of cancer risk and disease outcome. This review examines the current state of knowledge on and discusses the implications linked to telomere dysfunction and telomerase activation in the development and clinical outcome of HNSCC.

## Introduction

Head and neck cancer was expected to affect approximately 742,000 and 144,000 new patients, worldwide and in Europe, respectively, in 2015 [1]. The most common type of head and neck cancer is head and neck squamous cell carcinoma (HNSCC) which is a morbid, frequently lethal disease that develops in the epithelial cell lining of the upper aero-digestive tract (UADT, i.e., the oral cavity, pharynx, and larynx) [2]. While it has been strongly associated with tobacco use and heavy alcohol consumption, over the past two decades, high-risk alpha human papillomaviruses (HR *α-*HPV) have emerged as an important etiological factor for a subset of HNSCC arising from the oropharynx [3, 4]. The prevalence of HPV-driven HNSCC has been dramatically increasing in developed countries, predominantly affecting males at a younger age that those with tobacco- and alcohol-related carcinomas, and changes considerably across different geographical areas [5–7]. HPV-driven HNSCC and tobacco- and alcohol-related HNSCC are two biologically and clinically distinct entities [6, 8–14].

Primarily a locoregional disease at presentation [2], HNSCC frequently spreads to the neck lymph nodes, an event that usually occurs before it metastasizes to distant organs. Approximately one third of patients present with early stage squamous cell carcinoma (SCC) (small primary tumors with no evidence of lymph node metastases). This prognostically favored subgroup is generally treated with a single-modality approach consisting in surgery or radiotherapy alone, with cure rates reaching up to 90 % [15]. Most HNSCCs present, however, at an advanced stage, with the majority of patients facing combined modality therapy consisting in surgery followed by adjuvant radio-(chemo)therapy or upfront chemoradiotherapy with salvage surgery in non-responders [16, 17]. The extraordinary advancements in treatment strategies have not as yet produced the hoped for cure rates [18]; regrettably, the majority of HNSCC patients who show a complete response to locoregional treatment later develop locoregional recurrence, and approximately 35 and 30 %, respectively, develop distant metastases or second primary tumors of the UADT, lung, or esophagus [19, 20]. The median survival in patients with inoperable recurrent or metastatic disease is only 10 months in those receiving the best established chemotherapy regimen [21]. Intensive treatment strategies for locoregional advanced disease such as sequential or concurrent chemotherapy in addition to locoregional treatment, taxane-platinum combination chemotherapy regimens, and altered fractionation radiotherapy protocols have yielded encouraging results, although at the expense of substantial acute and late toxicity [22–24].

Traditional prognostic factors such as overall stage and neck metastases at presentation seem to have limited prediction accuracy and reproducibility [25]. In an era of biomarker-driven personalized cancer therapy, several investigations are currently examining new biological markers as prognostic and predictive factors in HNSCC [26]. But with the exception of positivity to HPV and its surrogate cyclin-dependent kinase inhibitor p16^INK4a^, no other prognostic biomarkers are presently being used in routine clinical practice in head and neck oncology [27].

HNSCC is a heterogeneous disease whose pathogenesis is a multistep, multifactorial process involving genetic and epigenetic aberrations [28]. Invasive HNSCC may be clinically preceded by visible pre-neoplastic lesions often appearing as white patches, particularly common in the oral cavity and larynx [29], exhibiting an annual transformation rate exceeding 3 % [30]. Termed leukoplakia, these white patches may harbor hyperplastic or dysplastic epithelial lesions and their molecular characterization may quantify the risk of transformation [31]. Genetic alterations in pre-neoplastic lesions of the UADT may reflect aberrations in cellular differentiation or cell cycle control. Suprabasal expression of low molecular weight keratins [29], loss of heterozygosity of the *TP53* gene [32], microsatellite instability [33], and higher chromosomal aneuploidy rates [34] all increase the risk of malignant transformation. Unfortunately, several pre-neoplastic lesions in the UADT are not clinically detectable.

Slaughter et al. first formulated the theory of “field cancerization” in 1953 [35] to explain high recurrence rates following tumor resections or UADT metachronous second primary tumors in patients treated for HNSCC. According to this model, the emergence of malignant lesions is preceded by the development of precancerous fields characterized by genetic alterations linked to carcinogen exposure. Following crucial genetic hits, a cell within the field can become cancerous and eventually give rise to invasive SCC. The risk of a second tumor is, moreover, markedly higher in cases in which this more-prone-to-transformation mucosa partially persists after the primary tumor has been treated, and this mechanism has recently been described in molecular terms [36, 37]. Clusters of cells with cancer-associated genetic alterations such as *TP53* mutations have been detected in biopsies of histologically normal mucosa of HNSCC patients, and, in particular, in those with multiple primary malignancies [38]. Proteomic analysis has recently detected abnormal proteomic profiling in tumor-adjacent and tumor-distant UADT mucosa samples without histological aberrations [39]. Identifying markers of field cancerization could, therefore, hold promise for improving risk assessment and personalized post-therapy surveillance in HNSCC patients.

Recent whole-exome sequencing studies have painted new pictures of the genetic landscape of HNSCC and have uncovered unexpected therapeutic targets [40]. HNSCC’s mutational landscape is dominated by tumor suppressor genes with activating oncogene mutations playing an additional relevant role [41–43]. *TP53*, *CDKN2A*, *CCND1*, *PIK3CA*, and *NOTCH1* are the most commonly mutated genes in HNSCC. Telomerase reverse transcriptase (*TERT)* promoter mutations resulting in increased telomerase expression have also been detected in a significant proportion of HNSCC patients [44–46].

The tumor suppressor p53 protein and the retinoblastoma (RB) tumor suppressor protein pathways are the most frequently deregulated signaling pathways in HNSCC [47]. Since activated p53 triggers the expression of several genes involved in cell cycle arrest, DNA repair, or apoptosis, it plays a crucial role in tumor suppression [48]. RB inhibits E2F transcription factor by direct protein-protein interactions thus preventing transition to the S phase of the cell cycle and promoting cell cycle arrest in G_1_ [49].

Most HPV-negative tumors harbor inactivating mutations in the *TP53* gene [50]. In HNSCC with wild-type *TP53*, the protein may be inactivated by binding to the HPV E6 oncoprotein or to the cellular over-expressed *MDM2* oncogene. Overall, the p53 pathway is down-regulated in at least 80 % of HNSCC [2].

The p16^INK4a^-cyclin D1-RB axis is also frequently deregulated in HNSCC. HPV-negative HNSCC show inactivation mainly by deletion or promoter hypermethylation of the CDKN2A gene encoding p16^INK4a^ [51] and frequently have CCND1 amplification [52], which encodes cyclin D1, with both leading to a decrease in the growth-suppressive hypo-phosphorylated RB form.

In HPV-driven HNSCC, the p53 and RB pathways are both inactivated at the protein level. The E6 protein promotes cell proliferation by stimulating ubiquitination and proteasome-dependent degradation of the p53 protein via the formation of a trimeric complex including E6, p53, and the cellular ubiquitin ligase E6AP. In addition to targeting p53, HR *α-*HPV E6 activates telomerase [53]. E7 viral oncoprotein targets the RB/E2F complex, resulting in the dissociation of RB-family proteins from E2F transcription factors and inducing S phase entry [54]. HPV-driven HNSCC shows, moreover, a higher incidence of activating mutations in the *PIK3CA* gene, fewer gross chromosomal aberrations, and approximately one half the mutation rate of its HPV-negative counterparts [43]. In addition, vascular endothelial growth factor (VEGF)-C and VEGF receptor 3 are involved in the molecular pathways that lead to newly formed intra- and peritumoral lymphatic vessels, thereby endorsing cancer cell diffusion to the regional lymph nodes and explaining the high propensity of HNSCC for neck node metastases [55].

Regardless of what the driving force in HNSCC carcinogenesis may be (HR *α*-HPV persistent infection or tobacco/alcohol-related alterations in the expression of oncogenes and tumor suppressor genes), an unlimited replicative potential continues to be the hallmark of cancer [56]. The telomere/telomerase interplay is a key element in determining genomic stability and cellular replicative potential [57], and both telomerase expression/activity and telomere dysfunction have been extensively investigated in human cancer [57–59] with most studies indicating that they are crucial, early events in tumorigenesis often detectable at the precursor lesion stage [60–65].

This review examines current knowledge on the implications of telomere dysfunction and telomerase up-regulation in the development and clinical outcome of HNSCC.

## Telomeres and telomerase: structure and regulation

### Telomeres

Specialized DNA structures located at the ends of chromosomes, telomeres are essential for stabilizing chromosomes by protecting them from end-to-end fusion and DNA degradation. In human cells, telomeres are composed of (TTAGGG)n tandem repeats with a 3′ single-stranded overhang [66]. Telomeres are associated with capping proteins of the shelterin complex. Shelterin proteins enable cells to distinguish their chromosome ends from DNA breaks and to repress DNA repair reactions as well as to regulate telomere-based telomere maintenance [67]. Shelterin consists of six interdependent core subunits: TRF1 and TRF2 (telomeric repeat-binding factors 1 and 2), TIN2 (TRF1-interacting nuclear factor 2), Rap1 (TRF2-interacting protein 1), POT1 (protection of telomeres 1), and TPP1 (POT1 and TIN2-interacting protein 1) [67]. TRF1 and TRF2 bind the double-stranded telomeric repeats, whereas POT1 is recruited to the single-stranded overhang through its specific single-stranded DNA-binding activity and its interaction with TPP1. TIN2 tethers TPP1/POT1 to TRF1 and TRF2 [68]. The single-stranded overhang forms the telomeric loop (T-loop) by invading the double-stranded TTAGG repeats. T-loop remodeling, which is promoted and maintained by the members of the shelterin complex, mainly TRF2, prevents the recognition of chromosome ends as sites of double-strand breaks, thus repressing DNA damage signaling pathways and classical non-homologous end joining at telomeres or homologous recombination [69]. In particular, TRF2 represses the ataxia telangiectasia mutated protein (ATM)-mediated DNA damage signal, while POT1 prevents the ataxia telangiectasia and Rad3-related (ATR)-mediated DNA damage response, and both prevent end-to-end telomere fusions [67, 70].

Since DNA polymerase is unable to completely replicate the 3′ end of chromosomes, the loss of telomeric repeats, which occurs at each round of DNA replication, progressively reduces telomeres to a critical length [71]. While random DNA damage activates a DNA damage response (DDR) until damage is removed, dysfunctional telomeres trigger persistent DDR activation [72]. Indeed, lacking key shelterin proteins, uncapped telomeres trigger a DDR resulting in cell cycle arrest, cellular senescence, and finally apoptosis, mediated by cell checkpoints, such as p53 and p16^INK4a^/RB signaling pathways [73].

TRF2 depletion predominantly leads to ATM activation, whereas deprotection of 3′ single-stranded overhang due to POT1 depletion activates ATR. ATM and ATR phosphorylate downstream targets [74], eventually leading to phosphorylation and p53 activation [75]. p53 induces transcription of the cyclin-dependent kinase inhibitor p21^CIP1^ to promote cell cycle arrest [76] and apoptosis by up-regulating several pro-apoptotic target genes [77].

When p53 and p16^INK4a^/RB signaling pathways are inactive, cells can bypass senescence and continue to proliferate, allowing further telomere erosion which leads to increased genomic instability, including chromosome breakage-fusion-bridge (BFB) cycles causing structural chromosomal rearrangements. This results principally in an additional proliferative constraint characterized by mitotic catastrophe with widespread cell death [78]. Telomerase or other mechanisms of telomere preservation can be stochastically re-activated in the rare cells that have entered the BFB process. In this scenario, telomeres are maintained and genetically unstable cells gain immortal growth capacity, a crucial event on the path towards malignancy [79].

**Fig. 1:**
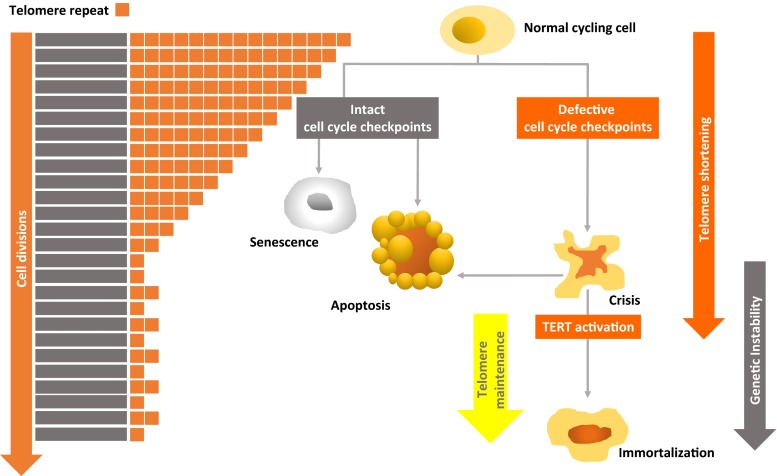
Short telomeres: senescence and cancer. Telomeres, which are essential to protect chromosomes from deterioration and from end-to-end fusion, are specialized DNA structures located at the ends of chromosomes composed of (TTAGGG)n tandem repeats that are associated with capping proteins. Human adult somatic cells have a limited capacity to divide (Hayflick limit) as DNA polymerase alone cannot replicate the 3′ end of the DNA strand resulting in a progressive loss of TTAGGG telomeric sequences. Critically short telomeres trigger a DNA damage response, resulting in cellular senescence, an efficient tumor suppressor mechanism, and apoptosis. Senescence involves intact DNA damage checkpoints, such as p53 and p16/RB signaling pathways. Checkpoint-compromised cells can escape cellular senescence and apoptosis. In this context, cells can experience an increased number of divisions and can ultimately enter to breakage-fusion-bridge events and dramatic genetic instability due to telomere erosion, which most commonly leads to cell death. Rarely, the cell reactivates telomerase expression to drive telomere maintenance and replicative immortality. *TERT* telomerase reverse transcriptase

Telomere/telomerase interplay is therefore critical in determining genomic instability and cellular transformation and, as such, each seems to be wearing the mask of Janus Bifrons, the two-faced Roman god. The consequence of telomere erosion depends, indeed, on the cellular milieu: in checkpoint-proficient cells, it leads to tumor suppression by senescence or apoptosis; while in checkpoint-compromised ones, it leads to tumor promotion by causing genetic instability (Fig. 1).

### Telomerase

More than 90 % of cancers acquire the capability to replicate indefinitely through telomerase expression [80], a ribonucleoprotein complex containing an internal RNA component (TR or TERC) and a catalytic protein, TERT, with telomere-specific reverse transcriptase activity [81]. Less than 10 % of human cancers, typically non-epithelial tumors, preserve their telomeres by a telomerase-independent alternative lengthening of telomeres (ALT) pathway [82].

TERT, which synthesizes *de novo* telomere sequences using TR as a template, is the rate-limiting component of the telomerase complex, and its expression is correlated with telomerase activity [81]. Expression of *TERT*, which is normally strongly repressed by multiple tumor suppressors and which plays a critical role in tumor formation and progression, is essential for unlimited cell growth and is under tight transcriptional control [83]. Regulation of telomerase operates at several levels: telomere-associated proteins, in particular TRF1 and POT1, are themselves negative regulators of telomerase as they repress telomerase access to telomeric ends [84]. Since longer telomeres contain larger amounts of TRF1, telomere length is usually maintained at stable average values in telomerase-expressing cells [85].

*TERT* gene transcription is probably the key determinant in telomerase activity regulation; more than 20 transcription factor-binding sites acting as activators or repressors have been identified within the *TERT* promoter. p53, which may be activated by telomere shortening, binds to transcription factor Sp1 and renders *TERT* core promoter inaccessible to *TERT* promoter activation in normal somatic cells [86]. Similarly, other cell cycle inhibitors such as p16^INK4a^ [87] and p27^KIP1^ [88] may down-regulate *TERT* expression. Nuclear factor-kB (NF-kB), hypoxia-inducible factor (HIF)-1, and the ETS/MYC complex bind to the *TERT* promoter thus increasing TERT expression. NF-kB can also induce TERT translocation from the cytoplasm to the nucleus [89]. *TERT* promoter activity is also critically dependent on the chromatin environment and DNA methylation status [90]. SET and MYND domain-containing protein 3 (SMYD3), a dimethyltransferase and trimethyltransferase, are significantly up-regulated in several cancers. SMYD3 increases trimethylation of histone H3-K4 in the *TERT* promoter, thereby directly trans-activating the *TERT* gene [91]. *TERT* promoter methylation status has unveiled a complex methylation pattern with some studies reporting hypomethylation in the CpG island in the *TERT* promoter region while others have described increased DNA methylation in TERT-expressing cancer cells [80].

Although telomerase’s primary function is telomere elongation and/or maintenance, several different extra-telomeric functions have also been described. These non-canonical functions may affect some cellular processes including gene expression, signaling pathways for the regulation of cell survival, resistance to stress, and apoptosis [92]. Of interest, the re-activation of telomerase in cancer cells may affect the cancer’s invasiveness and metastasis through the interaction of TERT with the Wnt/*β*-catenin [93] and the NF-kB signaling pathways [94].

In a minority of cases, cancer cells can maintain their telomeres by ALT. ALT-mediated telomere elongation is achieved by telomere recombination between telomere-sister chromatid exchanges or adjacent chromosomal telomere [95]. The ALT phenotype is usually characterized by heterogeneous telomere lengths, the presence of extra-chromosomal telomeric DNA molecules amassed within ALT-associated promyelocytic leukemia (PML) bodies, and reduced compaction of telomeric chromatin [96].

## Telomere dysfunction in HNSCC

In physiological conditions, the stratified squamous epithelium expresses low levels of telomerase activity [97]. Regulated telomerase activity has indeed been detectable in a subset of normal transit-amplifying stem cells residing in the basal layer providing them with an extended proliferative competence [98]. Consistently, non-cancerous epithelia show basal layers with longer telomeres with respect to parabasal and suprabasal ones [64].

Controlled telomerase expression is nevertheless inadequate to prevent telomere attrition during aging [99], and telomere shortening and DNA damage accumulate in human stem cells [100]. Furthermore, during suprabasal differentiation, telomerase expression is silenced *via* the recruitment of the RB-associated histone deacetylase repressor complex to the −98 E2F site of *TERT* promoter [101]. All epithelial cells may experience a progressive shortening of TTAGGG repeats that eventually results in replicative senescence, a state of growth arrest which is considered an effective tumor suppressor mechanism [76]. In human oral keratinocytes, senescence is associated with enhanced expression of discrete gene groups including G-protein-coupled receptors, matrix metalloproteinases (MMP), apolipoproteins, and mitochondrial proteins [102]. Senescent cells may acquire a senescent-associated secretory phenotype (SASP) characterized by increased secretion of pro-inflammatory cytokines such as interleukin (IL)-6, IL-1, and IL-8, matrix metalloproteinases, and reactive oxygen species [103]. SASP may create a pro-carcinogenic microenvironment in UADT, promoting tumor development, proliferation, and invasiveness [104].

As stated above, the abrogation of important cell cycle checkpoints, such as p53 and RB, may allow cells to bypass replicative senescence and to enter a state of crisis characterized by extremely short telomeres which may lead to BFB cycles and chromothripsis [105].

The presence of anaphase bridges in the majority of HNSCC is suggestive of their escape from replicative senescence [106]. Supporting the theory that genetic instability in head and neck carcinogenesis can be triggered by telomere dysfunction, telomere attrition has been associated with BFB events, accumulation of centrosomes, and multipolar cell divisions in cell lines from benign and malignant head and neck tumors [107, 108]. Also demonstrated in mice models, telomere attrition appears to be the main driver of genetic instability [109].

Findings indicating that telomere aberrations, mainly consisting in shortening, are consistently found in HNSCC precursors and in mucosa surrounding pre-neoplastic areas and invasive UADT carcinomas [63, 64, 110, 111] support the hypothesis that genomic instability driven by telomere dysfunction is an early event in the HNSCC oncogenetic process. Notably, telomere shortening has also been detected in nearby non-neoplastic esophageal epithelium from patients with invasive esophageal cancer [112] as well as in cancer-associated normal stromal cells and pre-neoplastic lesions, respectively, from the prostate [113] and the pancreas [114]. Telomere erosion is thus emerging as a very early, common genetic event in epithelial carcinogenesis [63]. Remarkably, recent whole-exome sequencing studies have disclosed that genomic instability is a dominant feature in HNSCC with about 80 % showing significant chromosomal instability and more than 40 % exhibiting whole genome duplication [115, 116].

Recently explained in molecular terms, field cancerization of UADT epithelium [117] offers a unique opportunity to study the multistep process of carcinogenesis during its earliest stages. Telomeres in the epithelium of the UADT from healthy subjects have been shown to shorten with age [118]. But the lengths of telomeres in histologically normal mucosa surrounding UADT cancer lesions do not appear to be correlated with patients’ ages, thus suggesting that the epithelium harbors some proliferative aberrations [63].

Studies examining telomere length in uninvolved, adjacent epithelium in carcinoma *in situ* of the oral cavity and in oral specimens from individuals without HNSCC have demonstrated that they are significantly shorter and the anaphase bridges are more abundant in the surrounding tissue of carcinomas *in situ* with respect to those in control specimens [64]. Telomere length analysis of orthokeratotic dysplasia, a precancerous condition frequently associated with SCC and of its surrounding background tissue, uncovered significantly shorter telomeres and a higher frequency of BFB with respect to those in controls [110].

A substantial number of HNSCC patients have been found to have extremely short telomeres in histologically normal mucosa surrounding SCC and, even more unexpectedly, short telomeres in background tissue were strongly and independently associated with mucosal failure [63]. It can be inferred from these findings that SCC arises in a telomere-shortened epithelial field characterized by genetic instability and a prone-to-transformation status. In this context, telomere shortening can be considered a biosensor of field cancerization that can identify patients at risk of local relapses or second field tumors. Supporting the hypothesis that telomere attrition precedes telomerase re-activation, TERT levels in mucosa surrounding HNSCC were not correlated with mucosal failure [63].

The amplification or re-activation of telomerase expression in stem or differentiated cells is considered a key event to escape crisis and to gain immortalization [97], and telomerase expression is necessary to convert and maintain an immortalized phenotype in human oral keratinocytes [119].

## Telomerase in HNSCC

While telomerase activity is detectable in most tumors, it is usually absent in normal somatic cells [66]. Telomerase activity can be measured by means of polymerase chain reaction (PCR)-based telomere-repeat amplification protocol (TRAP), by scoring immunohistochemical TERT staining or by quantifying *TERT* mRNA using real-time PCR [81, 120, 121].

When real-time PCR or TRAP have been utilized, weak telomerase expression and activity have frequently been found in normal UADT epithelium [61, 63, 122–136]. High levels of telomerase expression have been detected in as much as 75–100 % of HNSCC [111, 126–128, 130–135, 137–148]. According to *in vitro* studies, telomerase is induced in a substantial proportion of HNSCCs in telomerase-deficient keratinocytes and not by telomerase overexpression in telomerase-positive cells. This can be inferred by the high anaphase bridge index, dicentric chromosomes, and multipolar spindles, which indicate that these cells have experienced a period of critically shortened TTAGGG repeats before telomerase re-activation [106, 107]. Telomerase re-activation or up-regulation can be achieved by gene amplification, promoter mutations, *TERT* mRNA alternative splicing, epigenetic changes, or through post-translational processing [97].

Liu et al. [149] reported that TR amplification was one of the most frequent amplifications observed in laryngeal SCC with the rate rising progressively with the increasing severity of the lesions: none were, indeed, found in normal epithelium and 100 % in invasive cancer. Telomerase activity, however, is strictly correlated with TERT levels, while higher TR levels do not affect its catalytic properties [81, 150]. Comprehensive genomic characterization of HNSCC has frequently uncovered amplification of chromosome 5p, which encompasses *TERT* gene, in both HPV-positive and HPV-negative carcinomas [42, 151].

*TERT* promoter is transcriptionally silenced upon cellular differentiation. In cancer cells, *TERT* expression is induced by several cellular transcription factors including NF-kB [152], *β*-catenin [153], and c-Myc [154]. Conversely, wild-type *TP53* down-regulates *TERT* by forming a complex with the transcription activator Sp1 and, thus, inhibiting Sp1 binding to the *TERT* proximal promoter [86].

HR *α-*HPV E6 oncoprotein contributes to cell immortalization and transformation through both p53 degradation and telomerase activation. HPV-driven oropharyngeal SCCs express very high TERT levels [63]. The mechanisms underlying telomerase activation by E6 viral oncoprotein have not been completely elucidated. E6 can indirectly increase *TERT* expression by inducing p53 degradation. Furthermore, the increase in telomerase expression and activity in HPV-transformed cells could be the effect of E6-induced *TERT* transcription or post-transcriptional mechanisms. E6 from HR *α-*HPV can activate telomerase *via* degradation of NFX1-91, a transcription repressor of *TERT* [155]. In addition, HPV16 E6 physically and functionally interacts with telomerase complex and increases TERT catalytic activity [53]. Latent membrane protein 1 (LMP1) of Epstein–Barr virus may activate *TERT via* NF-kB and MAPK/ERK1/2 [156, 157]. In EBV-associated nasopharyngeal carcinoma, LMP1 enhances *TERT* expression and phosphorylation through the PI3K/AKT signaling pathway, and this makes the cells more resistant to irradiation [157].

Very recently, two prevalent and mutually exclusive mutations in the promoter of TERT (228C>T and 250C>T) emerged as the most frequently observed non-coding mutations in cancer and were associated with high levels of telomerase in multiple cancer types [158]. Notably, these mutations specifically promote *TERT* expression in telomerase-negative differentiated cell compartments, while their impact in telomerase-positive stem cell reservoirs appears to be neutral [150]. Thus, *TERT* promoter mutations uncouple cellular differentiation and telomerase silencing. These mutations result in generating *de novo* binding sites for transcription factors of the E26 transformation-specific family (ETS). In particular, the ETS GA-binding protein transcription factor *α* subunit selectively links the mutant form of the *TERT* promoter and increases *TERT* transcriptional activity [159]. More common in tumors derived from cells with low self-renewal rates, *TERT* promoter mutations have also been detected in a significant proportion of oral tongue [44, 45] and laryngeal SCCs [46]. SCCs of the base of the tongue, which are usually HPV-related, harbor, instead, wild-type *TERT* promoter [44]. HR *α-*HPV infection and *TERT* promoter mutations may be alternative mechanisms to up-regulate telomerase in HNSCC [44]. Remarkably, 228C > T and 250C > T mutations in the *TERT* promoter are more frequent in laryngeal tumors in smokers compared to that in non-smokers and are independently associated with poor overall survival (OS) [46].*TERT* promoter activity can be modified by a common polymorphism within the preexisting ETS2 binding site in the *TERT* promoter with patients with the rs2736098 variant and in particular those exposed to tobacco and alcohol having a lower risk of SCC of the oropharynx [160].

As histone deacetylase inhibitor FR901228 induces a significant increase in *TERT* expression in oral cancer cell lines, it is possible that the latter is epigenetically controlled in HNSCC through changes in DNA methylation or histone acetylation [161]. *TERT* regulation can also occur *via* post-translational protein modification [162]. Specific protein kinase C isoenzymes, namely *α*, *β*, *δ*, *ɛ*, and *ζ* (over-expressed in this malignancy), in HNSCC have been shown to regulate telomerase activity by phosphorylating TERT. This phosphorylation results in the interaction of telomerase and chaperone protein hsp90, an essential step for telomerase holoprotein integrity and enzymatic activity [163].

As telomerase expression is found in a large fraction of HNSCC, a subgroup of these tumors does not seem to require telomerase to maintain telomere, although the presence of Taq polymerase inhibitors and RNA degradation during specimen collection and processing cannot be excluded [133]. Human laryngeal cancer cell lines that survive after transfection with RNAi plasmid targeting *TERT* sequence show sustained proliferation and the presence of PML bodies suggesting that they are capable of employing an ALT pathway to maintain their telomeres [164]. This finding may pose a challenge if telomerase inhibitors are used to treat HNSCC.

As far as UADT pre-neoplastic lesions are concerned, telomerase expression is positively correlated with their severity in both oral and laryngeal SCC [122, 123, 134, 146, 147, 149, 165]. Interestingly, oral verrucous leukoplakia, an aggressive, premalignant lesion, exhibits telomerase activity levels approaching those found in invasive oral SCC [129]. Telomerase can be associated, alone or together with other markers, with a more aggressive phenotype and behavior in HNSCC. Several studies have, indeed, reported that *TERT* mRNA levels and telomerase activity are higher in poorly differentiated SCC [63, 126, 143, 146, 166–169], increase with tumor stage [63, 131, 142, 166], and are associated with lymph node involvement [63, 126, 131, 137, 139, 170] or extracapsular extension of lymph node metastases [125]. Numerous studies have analyzed telomerase’s ability *to predict* outcome (Table 1) [46, 63, 125, 127, 130, 136, 139, 140, 144, 171–177], and most have reported a correlation between increased telomerase expression and activity and a reduced response to treatment and a higher rate of regional and distant metastases ultimately resulting in poor clinical outcome [63, 125, 127, 136, 144, 171, 173–176]. A study examining a large sample of neoplastic tissues from 217 HNSCC patients reported that telomerase activity was an independent predictor of poor survival after adjustment was made for age, site, stage, histological grade, tumor depth, and extracapsular extension [125]. These results have been corroborated by studies focusing on other malignancies that have shown that telomerase activity correlates with progression and poor prognosis in lung [60], colorectal [62], breast [178], and prostate [179] cancers. These data are consistent with previous experimental observation and studies using *in vivo* mouse models demonstrating that a systemic injection of anti-telomerase ribozyme inhibiting telomerase activity significantly reduces metastatic progression in tumor-bearing mice [180, 181].

**Table 1:** Studies focusing on telomerase activity and prognosis in head and neck squamous cell carcinoma cited in this review

Authors and reference	Number of cases	Detection (assay)	Main findings
Ogawa et al., 1998 [134]	25 patients with oral and oropharyngeal SCC (biopsies before radiotherapy, at 4,10, and 20 Gy)	TA (TRAP)	Lower levels of TA correlate with better response to radiation therapy (*P* = 0.025)
Lee et al., 2001 [130]	46 oral SCC + 15 normal oral mucosa	TA (TRAP)	TERT correlates with TA (*P* < 0.001); lack of TA in normal mucosa; TERT useful marker for early detection of neoplastic cells
		*TERT* mRNA (RT-PCR)	No significant correlation of TA and *TERT* mRNA with rate of recurrence
Patel et al. 2002 [165]	110 HNSCC + matched adjacent mucosa 40 precancerous/benign condition	TA (TRAP)	TA in adjacent mucosa correlates with poor 2-year disease-free survival (*P* < 0.05)
Fabricius et al. 2002 [120]	20 tumor margin from 20 patients with HNSCC + 3 tissue samples from each of 20 additional patients, one from the carcinoma centre, the tumor margin, and one distant from the tumor	TA (TRAP)	No significant associations between TA and prognosis
Koscielny et al. 2004 [129]	80 HNSCC + matched adjacent mucosa	TA (TRAP)	No correlation between TA and local and regional recurrences and survival
Liao et al. 2004 [115]	217 HNSCC + matched adjacent mucosa	TA (TRAP by PCR enzyme immunoassay)	High levels of TA in 63.3 % cancer tissues and only 4.1 % of adjacent mucosa. TA in cancer tissues correlates with extracapsular extension of lymph node metastases (*P* = 0.005) and with overall survival (*P* = 0.008)
Eissa et al. 2005 [163]			
Samny et al. 2005 [162]	35 patients with laryngeal SCC: tissue from tumor core, tumor edge, surgical resection margin, and lymph nodes (if present)	*TERT* mRNA (RT-PCR)	Multivariate analysis showed TERT levels in tumor edges significantly correlated with overall survival (*P* = 0.04)
Luzar et al. 2005 [166]	40 laryngeal and 16 hypopharyngeal SCC	*TERT* mRNA (relative quantification by PCR based kit)	No correlation between level of *TERT* mRNA and overall survival
Freier et al. 2007 [161]	352 HNSCC	TERT (FISH)	*TERT* gene amplification less common in oral SCC than in pharyngeal and laryngeal SCC (*P* < 0.001); however, TERT expression more frequent in oral SCC than in pharyngeal and laryngeal SCC (*P* = 0.047)
		TERT (IHC)	No difference for overall and disease-free survival for HNSCC with increased TERT expression
Pannone et al. 2007 [164]	42 oral SCC + matched adjacent mucosa	*TERT* mRNA (RT-PCR) TERT (IHC)	Stage I patients with higher TERT expression had a lower survival rate (*P* = 0.04)
Chen et al. 2007 [160]	82 oral SCC + 116 oral epithelial dysplasia + 21 specimens of normal oral mucosa	TERT (IHC)	Higher nuclear TERT labeling scores significantly correlate with higher recurrence rate (*P* = 0.044) and shorter 5-year overall survival rate (*P* = 0.011)
Chen et al. 2008 [117]	31 laryngeal SCC + 31 matched adjacent mucosa	TA (TRAP by PCR enzyme immunoassay)	Higher levels of TA in tumor tissue significantly correlate with shorter overall survival (*P* < 0.05). No correlation between TA in adjacent mucosa and overall survival
Fabricius et al. 2009 [126]	40 HNSCC + 38 tumor-free surgical margin + 18 tumor-free distant from tumor	TA (TRAP) TERT (IHC)	The period without recurrence was slightly but statistically not significantly shortened in patients with higher TERT immunoreactive score (*P* = 0.138)
Qu et al. 2014 [44]	235 laryngeal SCC	TERT promoter mutations (pyrosequencing)	TERT C250T mutation was associated with worse survival of laryngeal cancer patients (*P* = 0.01)
Boscolo-Rizzo et al. 2015 [61]	139 HNSCC + matched adjacent mucosa	*TERT* mRNA (RT-PCR)	Higher TERT levels in cancer tissues significantly correlate with higher risk of regional failure (*P* = 0.045), distant failure (*P* = 0.067), and worse disease-specific survival (*P* = 0.037)

*FISH* fluorescent *in situ* hybridization, *HNSCC* head and neck squamous cell carcinoma, *IHC* immunohistochemistry, *PCR* polymerase chain reaction, *RT* reverse transcriptase, *SCC* squamous cell carcinoma, *TA* telomerase activity, *TERT* telomerase reverse transcriptase, *TRAP* telomere-repeat amplification protocol

The associations between high telomerase expression and activity and more aggressive phenotypes and progression in HNSCC may be ascribable not only to its ability to maintain telomere lengths in rapidly proliferating cells but also to TERT’s extra-telomeric functions and their interactions with other cancer-related signaling cascades, such as Wnt/*β*-catenin and NF-kB pathways [92, 182]. TERT’s extra-telomeric functions are implicated in regulating several cancer hallmarks including cell proliferation, angiogenesis, invasion, and metastasis [57]. Sustaining the roles of telomerase non-canonical functions in tumor invasiveness and progression in HNSCC, the instauration of ALT mechanisms to elongate telomeres in laryngeal cancer cells following telomerase inhibition, while maintaining a transformed phenotype, has been associated with less aggressive tumor features [164].

The Wnt/*β*-catenin pathway is a crucial regulator of the self-renewal property of normal amplifying adult stem cells, and its deregulation plays a critical role in abnormal cell proliferation and oral oncogenesis [183, 184]. Activation of the canonical Wnt signaling pathway results in the stabilization and nuclear translocation of *β*-catenin. In the absence of Wnt stimulus, cytoplasmic *β*-catenin is constantly targeted to the proteasome by the multiprotein destruction complex, which includes protein kinases CK1γ and GSK3β [185]. When activated, the Wnt/*β*-catenin pathway results in increasingly higher levels of *β*-catenin that eventually move to the nucleus and interact with LEF/TCF transcription factors thus promoting the transcription of target genes such as *MYC* and cyclin D1 [186]. Epithelial-to-mesenchymal transition (EMT), an essential step in cancer invasiveness and metastatic dissemination which is characterized by loss of cell-cell adhesion and the acquisition of migratory properties, is regulated by several proteins including *β*-catenin and vimentin [187]. TERT can stimulate the EMT program and induce an undifferentiated stem-like phenotype, a process associated with *β*-catenin signaling activation [93]. TERT binds *β*-catenin thus preventing its degradation, enhancing its nuclear accumulation and transcriptional activity, and regulates vimentin transcription in cooperation with *β*-catenin [93]. It has recently been observed that the overexpression of *TERT* in oral SCC is sufficient to induce a mesenchymal phenotype, and this is strictly related to the activation of the Wnt/*β*-catenin pathway. Silencing *TERT*, instead, leads to the inhibition of Wnt/*β*-catenin signaling and the suppression of EMT in oral cancer [188]. Interestingly, *β*-catenin is a known activator of *TERT* expression [153].

The NF-kB pathway regulates the expression of several genes including those involved in cellular proliferation, differentiation, and apoptosis. NF-kB is constitutively expressed in HNSCC and plays a crucial role as the modulator of the gene expression program associated with maintaining the malignant phenotype, invasiveness, and metastasis in SCC [189, 190]. It has been established that NF-kB regulates *TERT* expression by binding to a site 350-bp upstream from the translational start site. TERT nevertheless directly interacts with the NF-kB p65 subunit and regulates the expression of NF-κB target genes, such as *IL-6*, tumor necrosis factor (*TNF*)-*α*, *IL-8*, *MMP9*, commonly over-expressed in HNSCC [191]. IL-6 and IL-8 both induce EMT and promote metastasis in HNSCC *via* activation, respectively, of JAK-STAT3-SNAIL and AKT signaling pathways [192, 193]. In addition to IL-6, TNF-*α* also converges upon STAT3 up-regulating it. Moreover, TNF-*α* increases cell motility, migration, and invasion of human hypopharyngeal cancer cells by inducing TWIST expression, a basic helix-loop-helix transcription factor and dominant regulator of the EMT program in many solid tumors [194]. Some have hypothesized that TERT provides oral cancer cells with invasion capability by modulating *cathepsin D*, *MMP2*, and *MMP9* which, in turn, degrade the extracellular matrix and collagen IV, essential for basement membrane stability and integrity [195].

**Fig. 2:**
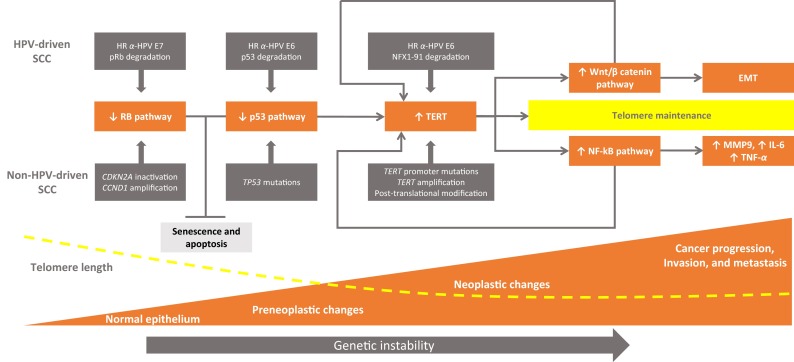
Epithelial carcinogenesis and telomere/telomerase interplay. The inactivation of the p53 and RB pathways are the main molecular determinants in head and neck carcinogenesis. In tobacco- and alcohol-related HNSCC, the abrogation of p53 and RB pathways may occur *via* mutation and genetic/epigenetic alterations. In HPV-driven carcinomas, p53 and RB pathways are inactivated at the protein level by E6 and E7 HR *α*-HPV oncoproteins, respectively. In this context, cells can bypass cellular senescence (a condition triggered by telomere shortening in which cells remain viable but are unable to divide) and experience an increased number of cell divisions of potentially premalignant clones characterized by extremely shortened telomeres and genetic instability. Different strategies may lead to re-activation of telomerase, a ribonucleoprotein complex containing an internal RNA component and a catalytic protein, TERT, with telomere-specific reverse transcriptase activity which synthesizes *de novo* telomere sequences. Cells can thus escape from apoptosis and maintain short but stable telomere lengths which are the key to cell immortality. Besides providing cells with unlimited proliferation potential, telomerase interacts with other cancer-related signaling cascades, such as Wnt/β-catenin and NF-kB pathways. In this scenario, telomerase plays additional non-canonical roles that may impact cancer progression by inducing crucial factors, such as MMP9, TNF-α, IL-6, and activating cellular programs leading to increased tumor cell motility/migration/invasion capability and epithelial-to-mesenchymal transition, in a context of feed-forward loops. *HR α-HPV* high-risk alpha human papillomaviruses, *TERT* telomerase reverse transcriptase, *NF-kB* nuclear factor-kB, *EMT* epithelial-to-mesenchymal transition, *MMP* matrix metalloproteinase, *TNF* tumor necrosis factor, *RB* protein retinoblastoma

HPV-driven oropharyngeal SCCs show a higher propensity for lymph node metastasis with respect to their HPV-negative counterparts and are not uncommonly characterized by an atypical pattern of distant metastases [6]. Preliminary data have shown that these malignancies may express extremely elevated TERT levels [63]. It can be speculated that the consequent increase in telomerase’s extra-telomeric functions may be related to this clinical behavior. TERT may, thus, be strictly involved in regulating critical SCC-related pathways, such as Wnt/*β*-catenin and NF-kB signaling, in a feed-forward loop context that amplifies and sustains autonomous cancer cell proliferation and tumor progression (Fig. Fig. 2).

## Telomere length in peripheral blood mononuclear cells (PBMC) and risk of HNSCC

As telomere length shortens with age, the parameter can be used as a marker of biological aging. But the high rate of inter-individual variations in telomere length among age-matched peers [196] appears to suggest that it reflects an individual risk for age-related diseases and cancer [197]. As telomere lengths in PBMC are strongly correlated with those in cells of different tissues, they are considered a surrogate marker for other tissues [198]. In cancer epidemiological investigations, telomere lengths in PBMC are usually determined to estimate the correlation between biological aging and cancer risk.

The role of telomere length in PBMC and cancer risk is quite controversial depending on the tumor type and the familial or sporadic context. Nevertheless, as suggested by Zhang et al. [58], various neoplasms show intrinsic biological heterogeneity, and different biological pathways may be modulated in various ways by telomere status. Telomere shortening in PBMC could be considered a biosensor of endogenous and environmental damage: factors increasing oxidative stress, e.g., cigarette smoking, are associated with telomere shortening in PBMC, while telomere erosion is, in turn, a driver of genetic instability which may promote tumorigenesis [58, 199, 200].

One large case–control study did not find a significant association between telomere length in PBMCs and HNSCC risk [201]. Conversely, two recently published case–control studies [202, 203] confirmed the results obtained a decade ago by Wu et al. [204] showing that telomeres were significantly shorter in PBMCs in HNSCC patients with respect to those in controls. When 266 patients with oral premalignant lesions or oral SCCs were compared with 394 age- and sex-matched controls, shortened telomeres were found to be an independent risk factor in PBMCs, although the risk was considerably higher in tobacco and alcohol consumers [202]. Telomere length also appeared to influence the malignant progression of pre-neoplastic lesions of the oral cavity. Short telomeres in PBMC may constitute an additional biomarker of oral habits and thus help to identify subjects at high risk of HNSCC. In another study [203] examining the association between telomere length in PBMCs and the risk of HR *α*-HPV-associated oropharyngeal SCC, the authors reported that short telomeres appeared to synergize with HPV type 16, increasing the risk of oropharyngeal SCC, particularly in the younger never smoker/drinker subgroup.

## Telomerase in the peripheral blood compartment: can liquid biopsies be used as potential biomarkers for HNSCC?

Circulating tumor cells and tumor-driven nucleic acids, such as circulating cell-free DNA and RNA, could be useful in detecting real-time tumor dynamics and in monitoring drug sensitivity during treatment.

Telomerase expression and activity in the peripheral blood compartment of HNSCC patients has been estimated in PBMCs or quantified by measuring circulating cell-free *TERT* mRNA by some studies.

The rate of telomerase activity in PBMCs has been found to be significantly higher in HNSCC patients with respect to that in healthy controls and associated with advanced stage, lymph node metastases, and poor overall survival (OS) [205, 206]. Two mechanisms have been hypothesized to explain this finding [205]. First, PBMC could be activated by soluble factors, secreted either by the cancer or by the tumor microenvironment. Mean serum vascular endothelial growth factor levels have, indeed, been significantly linked with telomerase activity in PBMCs in HNSCC patients [207]. Alternatively, PBMC could be activated after tumor antigen processing and cross-presentation has taken place in draining lymph nodes.

Several authors have reported higher *TERT* mRNA plasma levels in cancer patients with respect to those in controls, and a correlation has been found between circulating *TERT* levels and more severe clinical-pathological features and disease outcomes [208–210]; thus, circulating *TERT* could be considered a noninvasive tool for detecting cancers and monitoring the course of treatment. To our knowledge, the only study that has investigated the significance of *TERT* mRNA plasma levels in HNSCC patients reported that the values were indeed significantly elevated before surgery and that they decreased significantly two days after surgery [211]. Additional studies are clearly warranted to verify the feasibility of using cell-free circulating plasma *TERT* mRNA to diagnose the carcinoma early and to monitor treatment response in these patients.

## Conclusions

Although telomere dysfunction and telomerase activation appear to be dynamic processes during epithelial carcinogenesis, studies carried out until now have attempted to photograph them at a single point in time and have been unable to capture their mutable complex interaction. Our understanding of the complicated telomere/telomerase interplay in human cancer remains, in fact, for the time being incomplete. In the light of the comprehensive review of recently performed studies presented here, some conclusions can, however, be drawn about the clinicopathological and prognostic significance of telomere status and telomerase activity in HNSCC (Box 1).

Box 1. Key findings regarding telomeres and telomerase in HNSCC

**Table:** 

• HNSCC precursors and normal mucosa surrounding pre-neoplastic areas and invasive carcinomas are characterized by shortened telomeres.
• Short telomere lengths in mucosa surrounding HNSCC are strongly prognostic of mucosal failure.
• Short telomere lengths in normal mucosa surrounding HNSCC can be considered a marker of “field cancerization.”
• Telomerase activation plays a role in the majority of HNSCC cases.
• The timing of telomerase expression and activation may differ depending on the genetic and epigenetic context.
• Telomerase activity, which increases with tumor progression, is a prognostic marker of regional and distant failure.
• The significance of telomere length and telomerase activity in peripheral blood cells and of circulating *TERT* mRNA levels in the plasma of patients with HNSCC are complex issues warranting further studies

*HNSCC* head and neck squamous cell carcinoma, *TERT* telomerase reverse transcriptase

Analysis of SCC precursors and the normal mucosa surrounding pre-neoplastic and neoplastic areas has uncovered that extremely short telomeres are independently associated with mucosal failure and, thus, could represent potential biomarkers identifying patients at risk for relapses.

Several studies have shown that *TERT* mRNA levels and telomerase activity in HNSCC, which are associated with poor outcomes, gradually rise commensurately with the degree of epithelial aberrations and disease aggressiveness.

In conclusion, telomere/telomerase interplay and telomere shortening warrant further investigation in view of their ability to stratify HNSCC patients and the implications that they have on treatment and follow-up strategies in this particular patient population (Box 2).

Box 2. Questions that are still open with regard to telomeres and telomerase’s roles in the development of HNSCC

**Table:** 

• Are the mechanisms, significance, and effects of telomerase re-activation different in HPV-negative with respect to HPV-driven HNSCC?
• Are shortened telomeres in normal mucosa adjacent to HNSCC the consequence of greater cell proliferation or are they linked to an individual’s constitutive telomere length?
• How does telomerase interact and cooperate with other cancer-related intracellular signaling pathways in head and neck tumorigenesis?
• Are telomerase’s non-canonical functions critical for cancer invasion and metastasis HNSCC?
• What is the significance of telomere length in peripheral blood mononuclear cells?
• Is cell-free circulating plasma *TERT* mRNA a useful marker in diagnosing HNSCC and monitoring of treatment response?

*HNSCC* head and neck squamous cell carcinoma, *HPV* human papillomavirus, *TERT* telomerase reverse transcriptase
